# Effect of auditory neurosensorial stimulation in gait pattern after acute stroke

**DOI:** 10.1080/07853890.2021.1896544

**Published:** 2021-09-28

**Authors:** Marta Moiteiro, Ana João, Leonor Sacramento, Pedro Almeida, Ana Vidal, Ângela Maria Pereira, Sónia Vicente

**Affiliations:** a Department of Physiotherapy, Escola Superior de Saúde Egas Moniz (ESSEM), Egas Moniz Cooperativa de Ensino Superior, Caparica, Portugal; b Centro de Investigação Interdisciplinar Egas Moniz (CiiEM), Egas Moniz Cooperativa de Ensino Superior, Caparica, Portugal; c Hospital Garcia de Orta, Almada, Portugal

## Abstract

**Introduction:**

Auditory sensory stimulation is a therapy that influences cognitive, motor, emotional and behavioural functions and also patients’ quality of life. This approach is referred to be effective in optimising motor responses in neurological conditions [1]. When a stimulation occurs in the frontal lobe region of the brain it is possible to see through functional magnetic resonance imaging (fMRI) [2] that it trigger a response in motor regulation [3]. The purpose of this study is to investigate the effect of an auditory neurosensory stimulus (music) on gait pattern in post stroke individuals.

**Materials and methods:**

A cross sectional study was performed. Five patients (66.6 ± 14.1 years) with ischaemic stroke diagnostic participated in the study. Patients were assessed during the stance phase of gait cycle through 5-meter walking test, before and after the auditory neurosensory stimulus (music). STOMP was used to evaluate musical preference; cell phone camera to record gait pattern, speed and tibial angle in a lateral view and these data were analysed by Kinovea. All subjects signed an informed consent. This study followed all the principles of Helsinki Declaration.

**Results:**

Patients showed gait speed improvement (7.01 ± 0.94°) with auditory neurosensory stimulus (preference music). Data analysis in a lateral view of stroke limb by Kinovea showed better results in tibial angle in initial contact with stimulation (74.0 ± 8.0°) versus before stimulus (70.4 ± 11.0°). Also during mid-stance, results in toes/ground angle with stimulation (37.6 ± 16.1°) were higher than the results without stimulation (29.8 ± 14.9°)

**Discussion and conclusions:**

Stroke gait pattern observed in these patients changed with auditory neurosensory stimulation (preference music) which suggests increase of the responsiveness of the paretic ankle flexion. Some previous studies have shown that auditory neurosensory stimulation in stroke patient is efficient in the symmetry of the gait pattern and is a therapeutic advantage for motor recovery of the gait pattern [5]. This result enhances the importance of auditory stimulation (preference music) in a rehabilitation gait program for stroke patients. Nevertheless, only 5 patients were included, which is a limitation, so further studies are needed to extend these results.
